# Impact of parenting style disparities on mental health of medical college students: an analysis of mediating effects of positive psychological qualities

**DOI:** 10.3389/fpubh.2025.1584232

**Published:** 2025-04-28

**Authors:** Li Yang, Aili Song, Yanan Wu, Xusheng Wang, Yuting Liu

**Affiliations:** ^1^School of Medical Information, Wannan Medical College, Wuhu, China; ^2^College Student Mental Health Education and Counseling Center, Wannan Medical College, Wuhu, China; ^3^Department of Mechatronics and Automotive Engineering, Chizhou Occupational and Technical College, Chizhou, China

**Keywords:** mental health, parenting style disparities, positive psychological qualities, mediating effect, college & doctoral students

## Abstract

**Background:**

Research has shown that parenting style disparities have a significant impact on the mental health of college students. However, the underlying reasons behind this phenomenon have not been sufficiently explored. This study systematically examines the impact of parenting style disparities on the mental health of medical college students from the perspective of positive psychology and analyzes the mediating effects of positive psychological qualities.

**Method:**

This study administered questionnaires to 3,387 medical students in Wuhu, China, all of whom came from two-parent families. Specifically, the Short-Egna Minnen av. Barndoms Uppfostran for Children (s-EMBU-C) questionnaire was used to assess parenting style disparities, whereas the SCL-90 scale was utilized to evaluate mental health problems. Additionally, the Chinese College Student Positive Psychological Quality Scale was employed to assess positive psychological qualities. The hypothesis is proposed that positive psychological qualities mediate the relationship between parenting style disparities and mental health problems, and the bootstrap method was employed to examine the mediating effect. Additionally, a multivariate linear regression model was utilized to identify the factors associated with mental health problems.

**Results:**

The study revealed a significant positive correlation between parenting style disparities and mental health problems (*r* = 0.152, *p* < 0.01); conversely, parenting style disparities were significantly negatively correlated with positive psychological qualities (*r* = −0.070, *p* < 0.01). Additionally, positive psychological qualities were significantly negatively correlated with mental health problems (*r* = −0.201, *p* < 0.01). Furthermore, positive psychological qualities played a partial mediating role between parenting style disparities and mental health problems, accounting for 12.398% of the total effect.

**Conclusion:**

Parenting style disparities significantly negatively predict positive psychological qualities in medical college students, whereas positive psychological qualities significantly negatively predict mental health problems. Positive psychological qualities play a partial mediating role between parenting style disparities and mental health problems among medical college students. The main findings emphasize the importance of consistent parenting styles and the cultivation of positive psychological qualities for the mental health of medical college students.

## 1 Introduction

The mental health of college students primarily includes harmonious interpersonal relationships, stable emotions, objective self-assessment, and psychological adaptation (1). However, due to complex interpersonal relationships, academic pressures, and the challenges of job searching, many college students often experience mental health problems, such as depression, obsessive-compulsive behaviors, anxiety, and interpersonal sensitivity (2, 3).

With the rapid advancement of China’s economy and society, mental health problems among college students are becoming increasingly prominent. This is largely due to the intense competition they face and the need to navigate a more complex social environment.

The higher education enrollment rate rose from 30% in 2012 to 57.8% in 2021 (4). In March 2025, the Minister of Human Resources and Social Security announced that the number of university graduates in China is expected to reach 12.22 million, an increase of 430,000 compared to the previous year. This figure is projected to continue to grow over the next decade. The large population of university graduates exerts unprecedented pressure on the employment market and academia, requiring them to continuously enhance their skills to adapt to societal demands. However, this process is fraught with challenges, including anxiety, tension, and immense psychological stress.

The rapid rise of new media platforms in recent years, such as TikTok, Xiaohongshu, and Kuaishou, has also impacted the mental health of college students. According to the 2023 “College Student Social Media Usage Survey” conducted by China Youth Daily, more than 80% of college students frequently use social networks, and over half spend more than 3 h on social networking each day (5). However, an analysis based on the 2020 China Family Panel Studies data indicated that watching short videos significantly increases the risk of depression among young people (6).

A survey by the Institute of Psychology, Chinese Academy of Sciences, conducted in 2022 with approximately 80,000 students aged 15–26 revealed that 21.48% of participants were at risk of depression, whereas the anxiety risk was notably higher at 45.28% (7). The guidance and support of parents are crucial to college students in dealing with employment pressure, academic stress, and the influence of external environments, with parenting styles being a key factor in this process. Parenting styles influence children’s emotional development and coping abilities, which not only significantly impact their psychological health (8–11) but also play a crucial role in cultivating positive psychological qualities (12–15).

Baumrind (16) categorizes parenting styles as authoritative, authoritarian, and permissive, with the core differences lying in the balance between behavioral control and emotional support. Authoritative parenting is characterized by a “high-demand, high-responsiveness” approach, where parents establish clear rules while providing emotional support to help children internalize discipline (17, 18). This style has been confirmed as the most beneficial for child development across cultural contexts, as it fosters self-discipline, emotional regulation, and healthy parent–child relationships (19). Authoritarian parenting, on the other hand, adopts a “high-control, low-responsiveness” approach, where parents rely on coercive means to enforce rules while neglecting the psychological needs of the child (20). This unidirectional control model can lead to anxiety, aggression, and social withdrawal in adolescents, as the lack of emotional connection undermines the effectiveness of rule-based education (18, 21). Permissive parenting is characterized by a “low-control, high-responsiveness” approach, where parents may accept their children’s emotions but are lax in providing behavioral guidance. An excessively permissive environment can diminish adolescents’ self-discipline and increase the risks of substance abuse and bullying (18, 22).

An analysis of samples from 1,140 Chinese households across 25 provinces revealed that only 32% of families adopt the authoritative parenting style, whereas the combined proportion of authoritarian and permissive parenting styles is as high as 68% (23). These data reflect a significant structural bias in the choices of parenting styles among Chinese families today, as many parents have yet to fully realize the long-term impact of scientific parenting on child development.

This finding poses a significant challenge to the increasingly severe psychological pressure faced by college students in China. Therefore, this study explores the relationship between parenting styles and the mental health of student, to enhance parents’ awareness of the importance of their parenting approaches, which holds significant practical implications.

Research has indicated that a complex relationship exists between parenting style disparities, positive psychological qualities, and mental health. However, current studies have focused on the pairwise relationships between these factors, but the deeper connections among all three have not been explored. Exploring the complex interactions among these factors will aid in creating effective strategies to improve medical college students’ mental health.

To further explore the aforementioned relationships, we selected medical college students in China as the participants, as they face a longer academic journey and greater academic pressure than regular college students (2, 24). Additionally, as future healthcare professionals, they carry a greater sense of social responsibility (25). Therefore, maintaining good mental health is crucial for both the personal development and professional growth of medical students. This also underscores the significance of this research.

### 1.1 Impact of parenting style disparities on college students’ mental health

Previous studies have indicated that parenting styles have a critical effect on the psychological well-being of children (22, 26). However, most research has focused solely on the impact of either the mother or the father (27–29), but analysis regarding the differences in parenting styles between parents is lacking. Although a few researchers have found a strong correlation between the differences in parents’ educational approaches and children’s mental health, this area still requires further exploration.

Yaffe (30) conducted a study on the literature about the differences in parenting styles published between 1990 and 2020 from 15 countries around the world. The results revealed significant differences in parenting approaches between mother and father: mother exhibited a more authoritative parenting style than father, whereas father tended to be more authoritarian. These differences are influenced by a variety of factors, including social gender roles, educational level, socioeconomic status (SES), personality traits, and intergenerational transmission of parenting styles from grandparents.

Regarding social gender roles, both mother and father play different yet complementary roles in family education: mother are more nurturing, whereas father are more protective (31). For example, mother typically spend more time on household activities than father, which is closely related to the responsibilities of caring for children. Additionally, the social gender stereotypes and behavioral patterns of males and females also play a crucial role in this phenomenon (32). In China, Confucian culture has shaped a family gender preference for son over daughter, leading to significant gender differences in parenting styles among parents (33).

Parents with higher education degrees are more actively involved in their children’s academic development (34). They not only engage in in-depth discussions about plans with their children but also stimulate their educational aspirations through career enlightenment and life path design. Additionally, these parents emphasize the cultivation of positive learning qualities in daily interactions. Skilled at combining knowledge instruction with thinking training, they assist their children in establishing self-disciplined learning mechanisms while modeling the value of lifelong learning through their own behaviors.

Research has indicated that parents from high SES-families are more inclined to adopt supportive parenting styles, granting their children greater autonomy in decision-making and exerting less control (35). In contrast, parents from low-SES families tend to impose stricter rules, and exhibit more authoritarian traits, such as the frequent use of punitive measures and verbal threats, while demonstrating significantly lower levels of emotional engagement and involvement in daily interaction (36).

Dimensional analyses of parental personality traits, reveal that individuals with lower emotional stability (exhibiting significant neuroticism tendencies) often demonstrate pronounced negative affectivity. Such parents tend to prioritize their own emotional regulation over their children’s needs, manifesting specific behaviors like punitive discipline, emotional withdrawal, and excessive intervention (37). By contrast, parents with prominent agreeableness or open-mindedness exhibit greater emotional responsiveness in their parenting practices, characterized by acute sensitivity to children’s psychological needs and proactive support (38).

Parents under chronic stress are prone to developing emotional problems, such as anxiety, irritability, anger, and depression. These negative emotions significantly impair their capacity for child-rearing warmth, precipitating three maladaptive parenting behaviors: coercive disciplinary methods like corporal punishment, displaced emotional expressions projected onto children, and permissive neglect resulting from emotional exhaustion (39).

In addition, grandparents’ parenting styles have an intergenerational transmission effect on parents’ parenting styles, playing a significant mediating role in the transmission of personality traits, behavioral habits, and values between parents and their children (40).

Research has indicated that discrepancies in parenting styles play a significant role in explaining children’s developmental outcomes, even surpassing the contributions of the parents themselves (41, 42). Minor differences in parenting styles are suitable for adolescent development (43), but larger disparities in parenting approaches are associated with an increased risk of mental health and behavioral problems in children (44, 45). Zhen et al. (46) conducted a family survey among 2,598 medical college students, which revealed a positive correlation between differences in parenting styles and mental health problems. Liu et al. (13) confirmed that inconsistent parenting practices increase the risk of lifetime suicidal ideation.

Parenting style disparities are bound to lead to disharmony within the family. Such negative relationships can result in increased anxiety, depression, and other negative emotions for children, making them more susceptible to mental health problems (47–49). The family conflicts experienced by children can also be externalized, manifesting as aggression in interactions with peers (50). Consistent parenting philosophies can create a positive family atmosphere, improving children’s mental health (51). Such an environment provides children with a healthy and supportive growth setting, enhances their sense of security, alleviates negative emotions, reduces aggressive behavior, and further promotes the development of their mental well-being (52).

In conclusion, parenting style disparities significantly influence the mental health of college students. This study aims to explore how these differences in parenting styles impact medical college students’ mental health and to elucidate the underlying mechanisms involved.

### 1.2 Relationship between parenting style disparities, positive psychological qualities, and mental health

Positive psychological qualities refer to relatively stable positive psychological traits that individuals develop through the interaction of their innate qualities and environmental influences. These traits affect how individuals perceive issues, feel about them, and whether they adopt a proactive attitude towards problems, serving as the foundation for realizing an individual’s inner strength and potential (53).

The family parenting style has a significant impact on children’s positive psychological qualities. Positive parenting styles, such as warmth and understanding, have a beneficial effect on these qualities. Research has shown that parental emotional warmth is significantly related to various dimensions and overall scores of students’ positive psychological qualities (12). A regression analysis was conducted on parenting styles and positive psychological qualities, and the results revealed that maternal emotional warmth positively affects the formation of college students’ positive psychological qualities (13). Parents who provide understanding and establish appropriate boundaries encourage their children to independently solve problems, fostering confidence and independence. Conversely, negative parenting styles, like authoritarian and overly protective methods, impede the development of positive traits in children.

Inconsistent parenting styles are related to courage and temperance in positive psychological qualities and have a strong predictive power for adolescents’ courageous virtues, which can influence traits such as diligence, enthusiasm, and perseverance (14). Positive parenting styles, through emotional support and effective communication, can help children learn to regulate their emotions. Additionally, parental emotional support and positive feedback are significantly related to their children’s optimism (15). Authoritative parents offer emotional support and positive reinforcement, assisting their children in building a healthy self-image and fostering an optimistic perspective on the future. Additionally, by providing emotional support and encouraging their children to face challenges, parents can help them build resilience in coping with adversity (54). Parenting styles are crucial in shaping the positive psychological traits of children. Supportive parenting practices are vital in fostering children’s positive psychological qualities, such as optimism, gratitude, hope, resilience, a sense of meaning, and emotional regulation skills, through mechanisms of emotional support, behavioral modeling, rules and expectations, and communication and interaction. These qualities contribute to children’s psychological health.

Studies have found that positive psychological qualities have a significant influence and predictive power on mental health, with overall self-confidence demonstrating notable predictive power and impact on mental health (55). Personality traits such as resilience and tolerance can effectively act as buffers against stress and enhance individuals’ mental health (56). Positive psychological qualities are a strong indicator of college students’ mental health (57). In the emotional dimension, the persistence factor is a personality strength that helps prevent symptoms of depression and anxiety (58). In the interpersonal dimension, factors such as kindness, the ability to love, and social intelligence significantly predict positive mental health status (59). In the transcendental dimension, these qualities can significantly and negatively predict somatization and phobic factors in mental health assessment scales.

Positive psychological qualities, such as optimism and gratitude, can assist individuals in better regulating their emotions, and improvements in emotional regulation ability are closely related to better mental health (60). Positive psychological qualities, such as gratitude and kindness, can enhance individuals’ social relationships; those who are kind are more likely to receive help and support from others, and social support is an important protective factor for mental health (61). Positive psychological qualities, such as optimism and hope, can reduce the risk of mental disorders, such as depression and anxiety. Optimistic individuals tend to view the future positively and worry less about negative events, whereas hope instills confidence in the future and reduces feelings of helplessness. Additionally, positive psychological qualities, like curiosity, creativity, and openness, can promote individual self-growth and development. These qualities make individuals more willing to explore new things and accept challenges, leading to continuous psychological growth and progress (62).

Positive psychological qualities have a significant positive impact on mental health through various pathways, such as enhancing emotional regulation, promoting social support, fostering self-growth, increasing a sense of meaning and purpose, and improving coping strategies for stress. These qualities not only help individuals cope with life’s challenges but also contribute to the long-term development of their mental well-being.

In summary, we propose four hypotheses:

*Hypothesis 1*: A positive correlation exists between parenting style disparities and mental health problems among medical college students.

*Hypothesis 2*: A negative correlation exists between disparities in parenting styles and positive psychological qualities among medical college students.

*Hypothesis 3*: A negative correlation exists between positive psychological qualities and mental health problems among medical college students.

*Hypothesis 4*: Positive psychological qualities mediate the relationship between parenting style disparities and mental health problems.

## 2 Methods

### 2.1 Study participants and survey process

This study focused on students from Wannan Medical College in Wuhu, China, and applied a stratified cluster sampling technique to select the research sample. It encompassed all 32 majors at the college, including medical disciplines, such as clinical medicine, dentistry, and pharmacy, as well as non-medical fields, such as public administration, law, and applied psychology. Classes were taken as the units of investigation to ensure representation across all grades and majors. A total of 3,805 individuals participated in this research, and we retained the data of 3,387 students who came from two-parent families.

A total of three primary researchers were recruited, all graduate students majoring in psychology, who received training prior to the interviews. Following the principle of informed consent, questionnaires were administered and collected in classrooms on a class-by-class basis, with the testing taking approximately half an hour. During the administration, the primary researchers informed participants of the purpose and content of the tests, as well as the confidentiality of their personal data. After the students signed the informed consent forms, they filled out the paper questionnaires and returned them. No rewards were offered to any participants in this study.

### 2.2 Measures

#### 2.2.1 Parenting style disparities

The Parenting Styles Questionnaire, known as Egna Minnen av. Barndoms Uppfostran (EMBU) (63, 64), was originally developed by Swiss expert Perris to assess the parenting styles of both fathers and mothers, and it has demonstrated good reliability and validity. Arrindell et al. (65) distilled 46 items from the standard version of the EMBU to create the Short Egna Minnen av. Barndoms Uppfostran (s-EMBU). In this paper, we utilized the s-EMBU-C (66), which has been simplified based on the original s-EMBU to better suit the characteristics of Chinese students. This questionnaire consists of two versions: a paternal version and a maternal version, covering three aspects: rejection, emotional affection, and overprotection.

In s-EMBU-C, both the father and mother versions contain 21 items. For these items, each response is categorized as “never,” “occasionally,” and “frequently,” corresponding to the numerical values 1, 2, 3, and 4 in that order. Parenting style disparities are calculated using Formula 1, which is similar to those in references (46, 67). A key difference, however, is that its classification is not based on disparities in parental nurture–rejection, emotional warmth, or overprotectiveness.


(1) $$DPS=\overset{21}{\underset{i=1}{\sum }}|Fi-Mi|$$

where *i* represents the questionnaire items of s-EMBU-C, *Fi* denotes the score for the father’s parenting style for item, *i, Mi* represents the score for the mother’s parenting style for item *i*, and *DPS* denotes the value of the parenting style disparities. A larger score indicates a greater difference in parenting styles between the parents.

#### 2.2.2 Mental health problems

This study utilized Symptom Checklist 90 (SCL-90) (68), developed by L.R. Derogatis, to assess the psychological health status of university students. The scale consists of 90 items categorized into 10 factors: somatization, obsessive-compulsive symptoms, depression, anxiety, paranoia, psychosis, interpersonal sensitivity, phobia, hostility, and others. Each item is rated as none, mild, moderate, quite severe, and severe, corresponding to scores of 1, 2, 3, 4, and 5, respectively, in this study. The average score of the 90 individual items reflects the psychological health status, with a higher average indicating more severe psychological health problems. Generally, a total score exceeding 160, more than 43 positive items, or individual factor scores of ≥2 are considered positive detection criteria (69).

#### 2.2.3 Positive psychological qualities

The Positive Psychological Quality Scale for Chinese College Students (70) was used to assess positive psychological qualities. This scale consists of 62 items and can be divided into six dimensions: cognition, interpersonal relationships, emotions, justice, moderation, and transcendence. Each item is scored using a scale with the responses “very much like me,” “somewhat like me,” “neutral,” “somewhat unlike me, “and “very much unlike me.” In this study, these responses were converted into numerical values of 1, 2, 3, 4, and 5, respectively. The mean score of the 62 individual items reflects the level of positive psychological qualities, where higher average scores signify a greater extent of these positive qualities.

#### 2.2.4 Social-demographic characteristics

The social-demographic characteristics scale primarily gathers information on the participants’ gender, age, discipline, place of origin, and chronic disease status. Age is limited to integer values between 0 and 100. The responses for gender are “Girls” and “Boys;” for discipline, “Science” and “Liberal Arts;” for place of origin, “Countryside” and “City;” and for chronic disease status, “Yes” and “No.” The corresponding numeric values for these responses are 1 and 2.

#### 2.2.5 Parental characteristics

Parental characteristics comprise four items: parents’ occupations and educational levels. The answers for the first two items related to occupation are “Permanent job” and “Non-fixed job,” corresponding to numeric values of 1 and 2. For the last two items regarding education level, the answers are “Bachelor’s degree or above,” “Junior college or senior high school,” and “Junior high school or below,” corresponding to the numeric values of 1, 2, and 3, respectively.

### 2.3 Data processing

Data analysis was conducted using the web-based data science algorithm platform SPSSAU. Demographic information was described using frequency and percentage for each group, whereas the corresponding mental health problems were represented by their mean and variance. For all factors except age, one-way ANOVA was used to compare mental health problems across two or more groups. The age factor was examined using correlation analysis to investigate the connection between age and mental health problems. The bootstrap method was employed to examine the mediating effect of positive psychological qualities on the association between disparities in parenting styles and mental health problems. A multiple linear regression model was utilized to determine the factors linked to mental health problems. All tests were conducted using a two-tailed approach. A *p*-value of under 0.05 is regarded as statistically significant. Additionally, a 95% confidence interval (CI) for the mediating effect that excludes 0 implies a significant mediating effect.

To clearly display the differences among parenting style disparities, positive psychological qualities, and mental health problems in box plots, we normalized the values of parenting style disparities and positive psychological qualities. Before conducting the mediation analysis, we standardized the values of positive psychological qualities, parenting style disparities, and mental health problems to analyze the mediation effect more accurately. In all other cases, we used the original data for analysis.

## 3 Result

### 3.1 Social-demographic and parental characteristics

This study analyzed a sample of 3,387 medical students, with a mean mental health problems score of 1.502 ± 0.442. The criteria used to screen for individuals with psychological symptoms were a total score exceeding 160, a positive item count exceeding 43, or an average score greater than 2 for any individual factor. Among the participants, 26.60% were categorized as positive samples, while 73.40% were categorized as negative samples.

The ANOVA results indicated that gender (*F* = 6.307, *p* = 0.012), chronic disease (*F* = 21.531, *p* = 0.000), father’s occupation (*F* = 9.495, *p* = 0.002), and mother’s education level (*F* = 3.972, *p* = 0.019) are significantly associated with mental health problems. Correlation analysis revealed a linear negative correlation between age and mental health problems (*r* = −0.202, *p* = 0.000). For detailed information, please refer to Table 1.

**Table 1:** Descriptive analysis of mental health in Chinese medical college students (*n* = 3,387).

Variables	Mental health problems of medical college students(*n* = 3,387)					
	Total, *n*(%)	Mental health problems (mean ± SD)	*F*/*r*	*η^2^*	Cohen’s *f*	*p*
*n* (%)	3,387 (100)	1.502 ± 0.442	–	–	–	–
Mental health problems						
Positive	901 (26.60)	2.09 ± 0.39	*F* = 6204.27	0.647	1.354	0.000**
Negative	2,486 (73.40)	1.29 ± 0.20				
Gender						
Girls	1990 (58.75)	1.52 ± 0.44	*F* = 6.307	0.002	0.043	0.012*
Boys	1,397 (41.25)	1.48 ± 0.44				
Age	20.552 ± 2.429		*r* = −0.202	--	--	0.000**
Discipline						
Science	3,140 (92.71)	1.50 ± 0.44	*F* = 0.021	0.000	0.002	0.885
Liberal arts	247 (7.29)	1.50 ± 0.46				
Place of origin						
Countryside	1,603 (47.33)	1.51 ± 0.44	*F* = 1.116	0.000	0.018	0.291
City	1784 (52.67)	1.49 ± 0.44				
Chronic disease						
Yes	267 (7.88)	1.62 ± 0.51	*F* = 21.531	0.006	0.080	0.000**
No	3,120 (92.12)	1.49 ± 0.43				
Father’s occupation						
Permanent job	1,189 (35.10)	1.47 ± 0.43	*F* = 9.495	0.003	0.053	0.002**
Non-fixed job	2,198 (64.90)	1.52 ± 0.45				
Mother’s occupation						
Permanent job	1,661 (49.04)	1.49 ± 0.43	*F* = 2.682	0.001	0.028	0.102
Non-fixed job	1726 (50.96)	1.51 ± 0.45				
Father’s education level						
Bachelor degree or above	336 (9.92)	1.48 ± 0.44	*F* = 1.841	0.001	0.033	0.159
Junior college or senior high school	2,186 (64.54)	1.51 ± 0.44				
Junior high school or below	865 (25.54)	1.48 ± 0.44				
Mother’s education level						
Bachelor degree or above	210 (6.20)	1.42 ± 0.37	*F* = 3.972	0.002	0.048	0.019*
Junior college or senior high school	2,390 (70.56)	1.51 ± 0.44				
Junior high school or below	787 (23.34)	1.50 ± 0.45				

*p < 0.05, **p < 0.01.

### 3.2 Correlation analysis of parenting style disparities, mental health problems, and positive psychological qualities

The correlation analysis results revealed a significant positive relationship between parenting style disparities and mental health problems (*r* = 0.152, *p* < 0.01). Parenting style disparities were significantly negatively associated with positive psychological qualities (*r* = −0.070, *p* < 0.01), and positive psychological qualities were significantly negatively correlated with mental health (*r* = −0.201, *p* < 0.01). These findings suggest that the greater the parenting style disparities, the lower the positive psychological qualities, and the more severe the mental health problems. They also support *Hypotheses 1*, *2*, and *3*. The correlation coefficient matrix of positive psychological qualities, parenting style disparities, and mental health problems is listed in Table 2. In the table, “Positive” and “Negative” refer to the mental health problems of the samples. The criteria for classifying a sample as “Positive” include a total score exceeding 160, a count of positive items that exceeds 43, or an average score greater than 2 for any individual factor on the SCL-90.

**Table 2:** Descriptive statistics and correlation matrix for each variable (*n* = 3,387).

Variables	Mean ± SD			Parenting style disparities	Positive psychological qualities	Mental health problems
	Total	Positive (*n* = 901, 26.6%)	Negative (*n* = 2,486, 73.4%)			
Parenting style disparities	5.83 ± 3.728	7.33 ± 4.39	5.28 ± 3.29	1		
Positive psychological qualities	3.454 ± 0.730	3.06 ± 0.90	3.60 ± 0.60	−0.070**	1	
Mental health problems	1.502 ± 0.442	2.09 ± 0.39	1.29 ± 0.20	0.152**	−0.201**	1

*p < 0.05, **p < 0.01.

**Figure 1:**
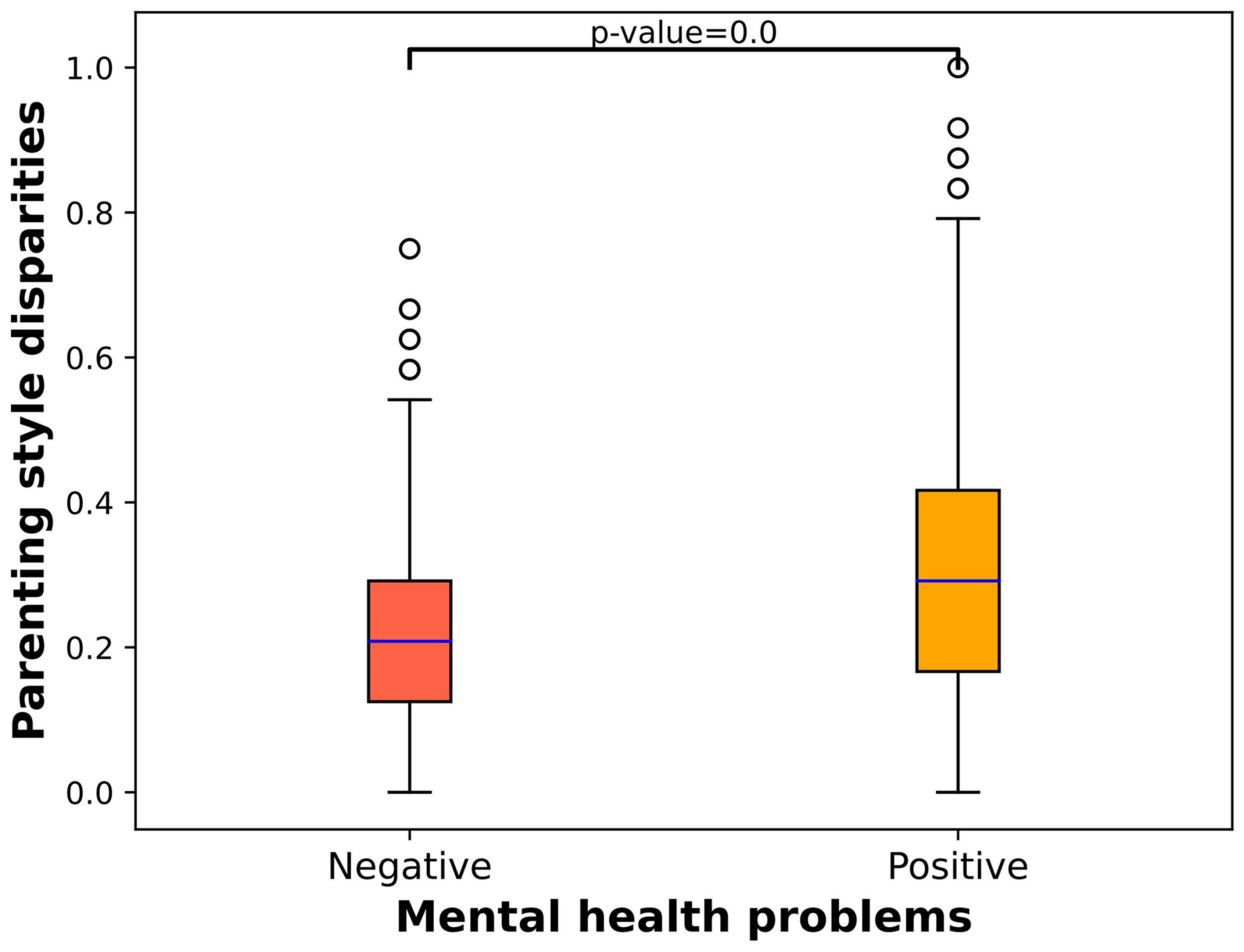
Box plot of parenting style disparities and mental health problems.

**Figure 2:**
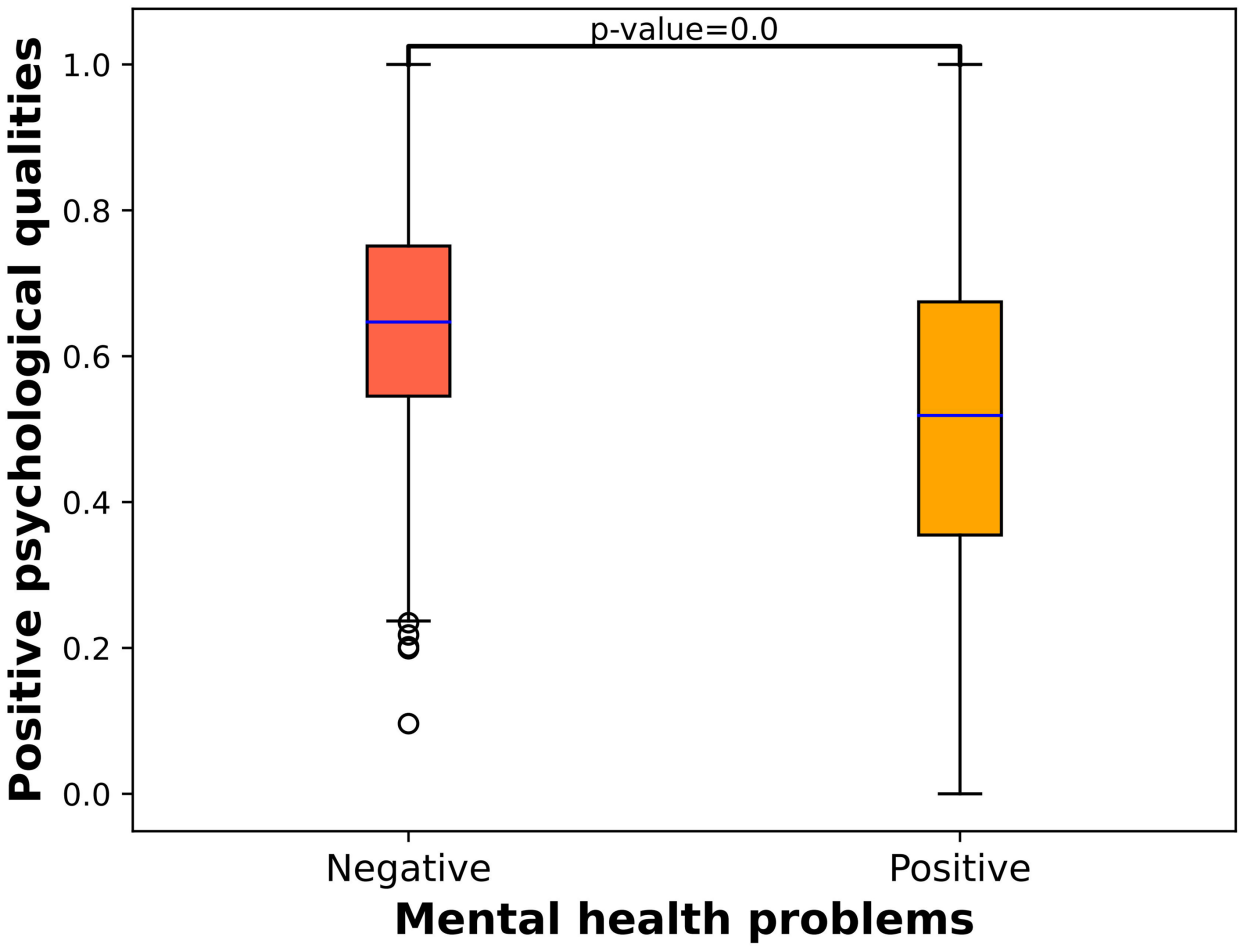
Box plot of positive psychological qualities and mental health problems.

We conducted a deeper analysis of the parenting style disparities between individuals with negative mental health problems and those with positive mental health problems as well as the variations in positive psychological qualities. Figure 1 illustrates the correlation between normalized parenting style disparities and mental health problems, showing that individuals experiencing positive mental health problems have significantly higher parenting style disparities than those experiencing negative mental health problems (7.33 vs. 5.28). Figure 2 displays the relationship between normalized positive psychological qualities and mental health problems, indicating that individuals with positive mental health problems exhibit significantly lower positive psychological qualities than those with negative mental health problems (3.06 vs. 3.60).

### 3.3 Mediating effects of positive psychological qualities between parenting style differences and mental health problems

To examine how disparities in parenting styles influence mental health problems, a bias-corrected bootstrap test (with 5,000 resamples) was employed, controlling for demographic factors like age and gender. Parenting style disparities were considered the independent variable, positive psychological qualities served as the mediating variable, and mental health problems were the dependent variable. The mediating effect of positive psychological qualities on the association between parenting style disparities and mental health problems was examined. For a more accurate comparison of the effect sizes among the variables, the data for each variable were standardized. A mediating effect is considered significant when the 95% CI for that effect excludes 0.

The findings of the mediation effect analysis are listed in Table 3. Parenting style disparities significantly and negatively predicted positive psychological qualities (*β* = −0.094, *p* < 0.001), with a mediation effect value of −0.094 and a 95% CI ranging from −0.128 to −0.060, excluding 0. Additionally, positive psychological qualities were found to significantly negatively predict mental health problems (*β* = −0.258, *p* < 0.001), with a mediation effect value of −0.258 and a 95% CI ranging from −0.290 to −0.226, which likewise does not include 0.

**Table 3:** Analysis of the mediating effect of positive psychological qualities on the relationship between parenting style disparities and medical college students’ mental health problems.

Model effect	Path	Effect value	Boot SE	BootLLCI	BootULCI	Proportion of effect	*p*
Total effect		0.196	0.017	0.163	0.229	100%	0.000**
Direct effect	Parenting style disparities=>Mental health problems	0.172	0.016	0.139	0.203	87.602%	0.000**
Indirect effect	Parenting style disparities=>Positive psychological qualities=>Mental health problems	0.0243	0.006	0.016	0.037	12.398%	0.000**
X=>M	Parenting style disparities=>Positive psychological qualities	−0.094	0.017	−0.128	−0.060		0.000**
M=>Y	Positive psychological qualities=>Mental health problems	−0.258	0.016	−0.290	−0.226		0.000**

*p < 0.05, **p < 0.01.

**Figure 3:**
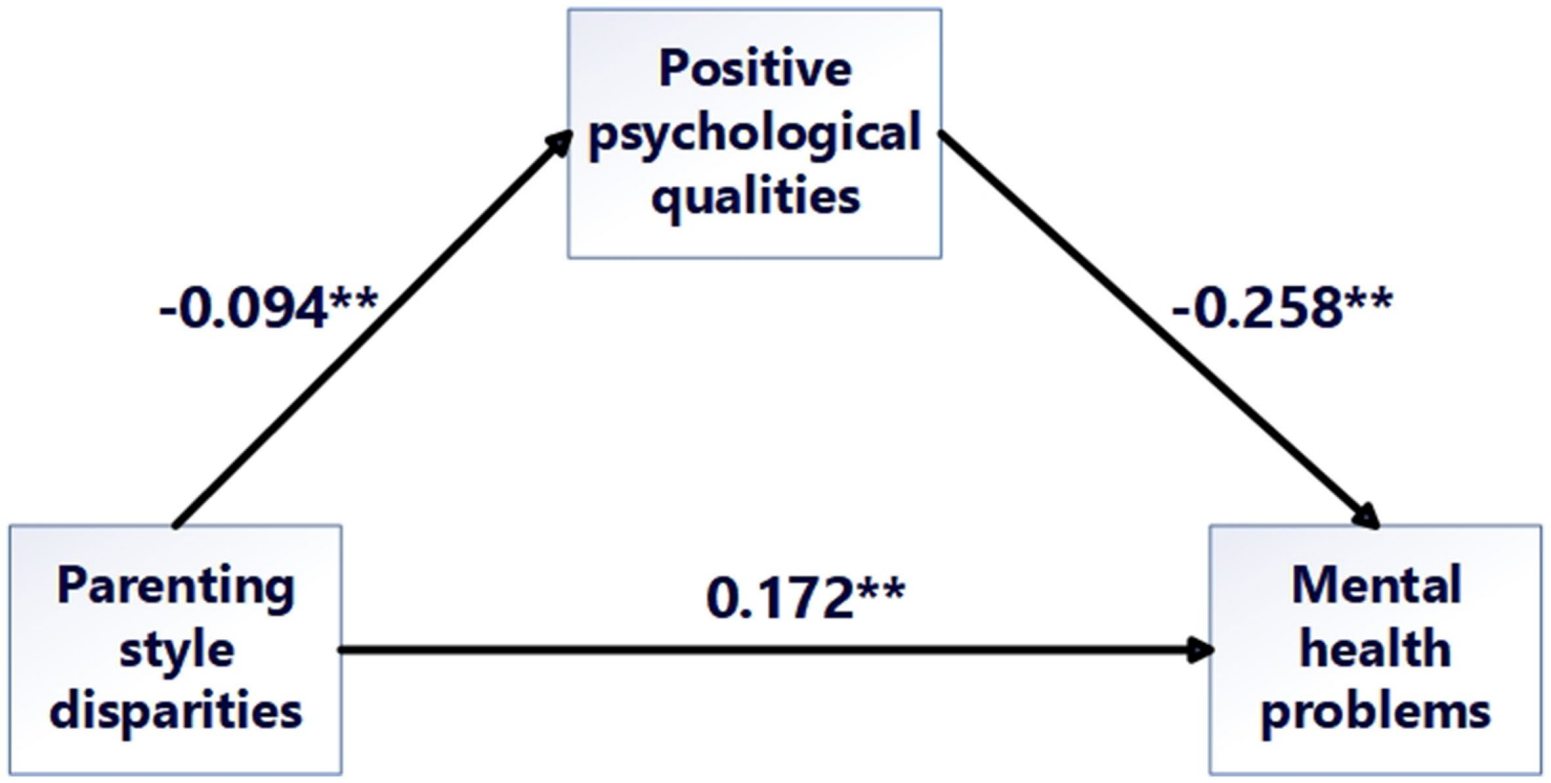
Mediating role of positive psychological qualities in the relationship between parenting style disparities and mental health problems among medical college students.

The direct effect value from parenting style disparities to mental health problems was 0.172, with a 95% CI of [0.139, 0.203]. The mediation effect value was 0.024, with a 95% CI of [0.016, 0.037], and the total effect value was 0.196, with a 95% CI of [0.163, 0.229]. All 95% confidence intervals exclude 0, indicating that both the direct and indirect effects of differences in parenting styles on mental health problems are significant. The direct effect accounts for 87.602% of the total effect, whereas the mediation effect accounts for 12.398%. These results suggest that positive psychological qualities play a partial mediating role in the association between differences in parenting styles and mental health problems, thereby supporting *Hypothesis 4*, as depicted in the mediation model presented in Figure 3.

### 3.4 Multivariate linear regression analysis of the association among parenting style disparities, positive psychological qualities, and mental health problems

In Table 4, the multivariate linear regression model identifies key factors related to mental health problems. The results reveal that mental health problems are significantly related to several variables: age (*β* = −0.034**), chronic disease (*β* = 0.115**), father’s occupation (*β* = 0.041*), parenting style disparities (*β* = 0.019**), and positive psychological qualities (*β* = −0.150**).

**Table 4:** Multivariate linear regression analysis of mental health problems among Chinese medical college students (*N* = 3,387).

Independent variables	Regression coefficient	95% CI	Multicollinearity diagnosis	
			VIF	Tolerance
Constant	2.489**(24.347)	2.289 ~ 2.690	–	–
Gender	−0.017(−1.192)	−0.045 ~ 0.011	1.016	0.984
Age	−0.034**(−11.506)	−0.039 ~ −0.028	1.013	0.987
Discipline	0.012 (0.432)	−0.042 ~ 0.065	1.012	0.988
Place of origin	−0.006(−0.440)	−0.034 ~ 0.022	1.019	0.982
Chronic disease	0.115**(4.395)	0.064 ~ 0.167	1.009	0.991
Father’s occupation	0.041*(2.398)	0.008 ~ 0.075	1.359	0.736
Mother’s occupation	−0.011(−0.461)	−0.058 ~ 0.036	2.871	0.348
Father’s education level	−0.010(−0.809)	−0.034 ~ 0.014	1.017	0.984
Mother’s education level	−0.006(−0.811)	−0.022 ~ 0.009	2.370	0.422
Parenting style disparities	0.019**(9.838)	0.015 ~ 0.022	1.015	0.985
Positive psychological qualities	−0.150**(−15.494)	−0.170 ~ −0.131	1.016	0.984
*R^2^*	0.145			
Adjusted *R^2^*	0.143			
*F*	*F*(11,3,375) = 52.226, *p* = 0.000			

The dependent variable is the value of mental health problems, and the Durbin–Watson statistic (D–W) value is 0.278. VIF denotes “variance inflation factor.” *p < 0.05, **p < 0.01 (the values in parentheses represent *t*-values).

## 4 Discussion

### 4.1 Relationship between mental health problems and parenting style disparities among medical college students

This study revealed that the prevalence of mental health issues among medical college students was 26.60%, with an average score of 1.502 ± 0.442, which is slightly above the national norm of 1.47 ± 0.48 (71). Medical students who are younger, have chronic illnesses, or whose fathers hold unstable jobs are particularly vulnerable to mental health problems.

Parenting style disparities yield an average score of 5.83 ± 3.728, which significantly and positively predicts mental health problems, aligning with previous research findings (46, 67). In addition, Zhukova et al. (72) surveyed of 306 Russian families, demonstrating that incongruence between parental styles was significantly associated with children’s mental health problems. Consistent with this finding, References (43, 73) also reached the same conclusion.

The impact of varying parenting styles on the mental health of medical college students can be linked to two factors.

First, these disparities can foster family disharmony. A lack of effective communication between parents may lead to conflicts, negatively impacting family cohesion. A harmonious family environment is essential for college students, as studies have indicated that students from less harmonious family backgrounds are more prone to anxiety (74).

Second, differing parenting styles can result in cognitive dissonance. College students may receive conflicting information and expectations due to the contrasting approaches of their parents (75). For instance, one parent may adopt a permissive style with low demands and high responsiveness while the other employs an authoritarian approach with high demands and low responsiveness. This conflicting guidance can confuse children, impairing their self-perception and development of a clear identity and confidence.

However, this study did not delve into the direction of differences in parenting styles. Low consistency in parenting styles may be adaptive for adolescent development, and such differences do not necessarily imply unreasonable parenting practices (43). Whether children benefit may depend on the specific composition of these differences (72). Simons et al. (45) indicated that the combination of an authoritative parenting style by the mother and a permissive parenting style by the father can effectively reduce children’s crime rates and levels of psychological depression. Therefore, compared to disparities in parenting style, the direction of parenting style disparities may be even more important for student development, and this will be the focus of our next research endeavor.

### 4.2 Mediating effect of positive psychological qualities

The average score for positive psychological qualities was 3.454 ± 0.730, aligning with the findings of Fang (76) and Lin (77). The present study indicates that positive psychological traits act as a partial mediator in the relationship involving differences in parenting styles and the mental health of medical college students.

The indirect effect of positive psychological qualities on the relationship between parenting style disparities and mental health problems of college students is significant but modest (12.398%). Therefore, exploring the practical implications of this finding is crucial. To explore the practical implications of this finding, we consider two aspects.

On one hand, although no identical studies have previously been conducted, a similar study on impoverished students found that psychological capital partially mediated the relationship between negative parenting styles and mental health, with an indirect effect of 16.24% (78), which is close to our result.

On the other hand, with the development of positive psychology research, scholars are increasingly inclined to examine positive psychological qualities within specific cultural and social contexts, emphasizing a dialectical interpretation of happiness. Mayer et al. (79) highlighted that the life experiences of outstanding individuals are complex, with negative and positive emotions being intertwined, and negative emotions fostering positivity and creativity. Lomas et al. (80) revolutionized positive psychology by adopting a multifaceted perspective, highlighting the cultural and social environments behind individuals and seeking to understand them from a macro-perspective. This indicates that the causes of positive psychological qualities are extremely complex, and are not only related to differences in family parenting styles, as studied here, but also to regional culture and the social environment.

Given the significant impact of positive psychology on mental health (81), despite the modest indirect effect of positive psychological qualities at 12.398%, it is sufficient to underscore its importance.

**Figure 4:**
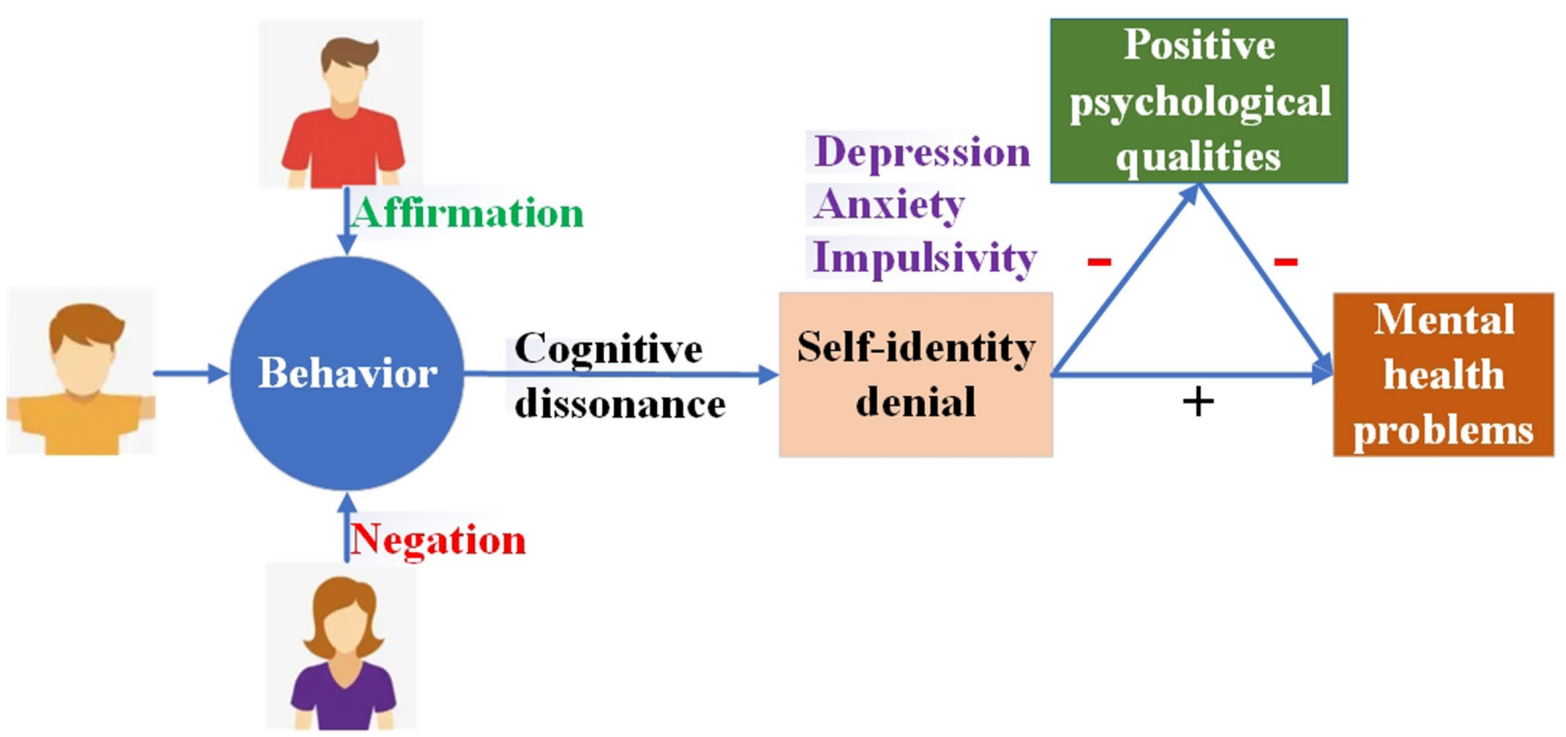
Parenting style disparities affect mental health via cognitive dissonance.

Cognitive dissonance theory may help to partially explain the mediating role of positive psychological qualities in the relationship between parenting style disparities and the mental health problems of college students. As shown in Figure 4, when differences in parenting styles are significant, parents often exhibit opposite attitudes toward their children’s behavior, with one parent affirming and the other negating. Eventually, this is bound to cause cognitive dissonance in the children. The most direct consequence of cognitive dissonance is the negation of self-identity. Individuals undergoing a self-identity crisis are more prone to negative psychological states, such as depression, anxiety, and impulsivity (82), which directly affect their mental health. Furthermore, from a positive psychology perspective, self-identity is crucial in the development of positive psychological qualities (62, 83). A strong sense of self-identity enhances an individual’s positive psychological traits, whereas a weak sense diminishes them. Accordingly, lower levels of positive psychological qualities imply that students have less psychological capital to effectively cope with various psychological challenges, making them more susceptible to psychological problems.

Seligman (62) asserted that, “Virtues and strengths are the core of an individual’s positive qualities. They can help overcome psychological disorders.” Positive psychological traits, such as hope, confidence, resilience, and compassion, are essential psychological assets that enable individuals to maintain a constructive mindset and effectively navigate various psychological challenges. Numerous studies have demonstrated a significant relationship between positive psychological qualities and mental health, highlighting that individuals with more pronounced positive qualities are at a lower risk of severe mental health problems (84, 85).

### 4.3 Impact of sociodemographic factors on mental health of medical college students

We observed that gender, age, chronic disease, and the father’s occupation are associated with the mental health of college students. These findings are consistent with previous research (86, 87), highlighting the need for increased attention to mental health issues in this population.

Females, in particular, tend to be more emotional, experience greater mood fluctuations, and are more susceptible to stress, resulting in typically more severe mental health problems than males (88).

The multivariate linear regression model presented in Table 4 reveals that age significantly and negatively predicts mental health problems (*r* = −0.034, *p* < 0.001). As students age and gain life experience, their social adaptability and psychological resilience tend to improve (67).

Additionally, underlying health conditions may impose extra stress during social interactions and academic pursuits, leading to negative emotions that adversely affect mental well-being (89).

Furthermore, children whose fathers hold stable jobs generally exhibit better mental health than those whose fathers lack employment stability. A steady father’s occupation often correlates with a reliable family income and higher socioeconomic status, which can positively influence the mental health of the children (90).

## 5 Limitations

First, this study employs a cross-sectional design, which does not permit causal inferences between variables; therefore, future research should consider utilizing longitudinal approaches. Specifically, researchers could track data changes within the same group of students over their college careers, divided by semester. For example, students could be categorized into groups based on the degree of parenting style disparities, allowing for the observation of how positive psychological qualities and mental health evolve within each group. Analyzing and comparing the data across these groups will yield more reliable conclusions.

Second, the data for this study were gathered using self-report methods, which may introduce the potential for social desirability bias. To improve the reliability and validity of future research, data should be obtained from teachers, parents, and peers. For example, data on the Disparities in parenting styles can be obtained through reports filled out by both students and their parents; data on students’ mental health issues can be obtained from reports filled out by the students themselves, peers, teachers, and their parents. By comprehensively considering reports from all parties, we can arrive at more fair and reasonable data.

Third, the generalizability of the research findings is limited, as the current study focuses exclusively on medical students. To enhance the academic value and applicability of the results, future research should include participants from nonmedical student populations. This expansion will facilitate a more comprehensive understanding of the issues and allow for a better assessment of the findings across diverse educational contexts.

## 6 Conclusion

This study confirms *Hypotheses 1, 2*, *3*, and *4*, indicating that differences in parenting styles positively predict the mental health problems of medical college students, with positive psychological qualities serving as a mediating factor. Measures can be taken to reduce disparities in parenting styles and enhance the positive psychological qualities of medical college students, thereby promoting their mental health development, by focusing on fostering family culture, eliminating gender discrimination, leveraging the exemplary role of parents, and establishing a home-school collaboration mechanism.

To cultivate a unified and positive family culture, parents should collaborate with their children to establish family rules and hold regular family meetings, fostering mutual understanding among family members. Consistency should be maintained in behavioral norms, thought patterns, and values, thereby forming a cohesive family culture. Parents should adopt parenting approaches that provide warmth and emotional support, thereby nurturing the development of positive qualities, such as optimism and resilience, in their children.

Parents should adhere to the principle of gender equality, attributing their children’s abilities and performances to effort rather than gender traits. They should document their daily parenting interactions and reflect on whether their behaviors remain neutral, avoiding gender biases in their interactions with their children, thus reconstructing equitable parenting practices.

Parents can serve as role models by embodying traits such as humility, courage, optimism, and resilience in their daily lives, thereby instilling these qualities in their children. Furthermore, the use of diagnostic educational assessments is advocated to monitor the state of individuals’ positive psychological qualities, enabling targeted educational interventions.

Establishing a home-school collaboration mechanism, the school regularly provides feedback to parents about students’ behavior at school. Parents actively participate in home-school education activities, thereby forming an educational cycle, ensuring smooth communication channels, and enabling early detection and resolution of students’ undesirable behavior.

## Data Availability

The raw data supporting the conclusions of this article will be made available by the authors, without undue reservation.
